# Implementation of fracture risk assessment in men with prostate cancer requiring long-term androgen deprivation therapy: a systematic scoping review using the i-PARIHS implementation framework

**DOI:** 10.1007/s11764-024-01659-3

**Published:** 2024-08-14

**Authors:** Qizhi Huang, Caroline Mitchell, Elisavet Theodoulou, Andrew C. K. Lee, Janet Brown

**Affiliations:** 1 Population Health, School of Medicine and Population Health, University of Sheffield, Sheffield, UK; 2 Faculty of Medicine and Health Sciences, Keele University, Keele, UK; 3 Clinical Medicine, School of Medicine and Population Health, University of Sheffield, Sheffield, UK

**Keywords:** Prostatic cancer, Androgen deprivation therapy, Osteoporosis, Cancer survivorship, Implementation, Primary health care

## Abstract

**Purpose:**

Androgen deprivation therapy (ADT) is a mainstay of treatment for prostate cancer (PCa) and is associated with increased risks of osteoporosis and fragility fractures. Despite international guidelines to mitigate fracture risk, osteoporosis is under-diagnosed and under-treated due to poor implementation. This scoping review aims to synthesise knowledge surrounding the implementation of guidelines to inform health service interventions to reduce fracture risk in men with PCa-taking ADT (PCa-ADT).

**Method:**

Four databases and additional literature were searched for studies published between January 2000 and January 2023. Studies that provided evidence influencing guidelines implementation were included. The i-PARIHS (Promoting Action on Research Implementation in Health Services) implementation framework was used to inform the narrative synthesis.

**Results:**

Of the 1229 studies identified, 9 studies met the inclusion criteria. Overall, an improvement in fracture risk assessment was observed across heterogeneous study designs and outcome measures. Six studies were from Canada. Two studies involved family physicians or a community healthcare programme. Two studies incorporated patient or specialist surveys. One utilised an implementation framework. Implementation barriers included the lack of knowledge for both patients and clinicians, time constraints, unsupportive organisational structures, and challenges in transferring patient care from specialists to primary care. Effective strategies included education, novel care pathways using a multidisciplinary approach, incorporating a healthy bone prescription tool into routine care, point-of-care interventions, and bespoke clinics.

**Conclusion:**

There is an unmet need to provide evidence-based bone healthcare in men with PCa receiving ADT. This study highlights barriers and strategies in the implementation of fracture risk assessment for PCa-ADT patients.

**Implications for Cancer Survivors:**

Primary care clinicians can play a significant role in the management of complications from long-term cancer treatment such as treatment-induced bone loss. Future studies should consult patients, families, specialists, and primary care clinicians in service re-design.

**Supplementary Information:**

The online version contains supplementary material available at 10.1007/s11764-024-01659-3.

## Introduction

Prostate cancer (PCa) is the most common cancer in men in over 100 countries [1]. In the UK, about one in eight men will be diagnosed in their lifetime [2]. Androgen deprivation therapy (ADT) is the mainstay of treatment for locally advanced or metastatic PCa, usually alongside anti-androgens and chemotherapy [3–6]. It is used on at least one-third of patients [4], and some may remain on it for up to two decades [5].

The initiation of ADT results in rapid and profound suppression of male hormones. While ADT is effective in reducing tumour growth, it also brings a range of complications including reduced bone mineral density (BMD), osteoporosis, sarcopenia, and impaired balance, causing an increased risk of falls and fractures [7, 8]. Fragility fractures can cause substantial pain, severe disability, and a reduced quality of life [9]. The mortality rate is also higher in men compared to women following a hip fracture [10]. The direct costs of overall fragility fractures in the UK population were €5.4 billion in 2019 accounting for 2.4% of healthcare spending in the country [11].

The identification of patients at high risk of fracture through prompt fracture risk assessment/BMD testing and the provision of bone protective medicine is effective in reducing bone density loss for patients with PCa taking ADT (PCa-ADT) [12] and is currently recommended in various international guidelines [13–15]. However, real-world data demonstrates that the implementation of these guidelines is poor [16, 17]. A UK hospital audit performed a decade ago and our recent study in primary care showed that fracture risk assessment and BMD measurement were not performed routinely in PCa-ADT patients in the UK [18, 19]. As PCa survival improves, many men require prolonged ADT. Consequently, the management of cancer treatment-induced long-term complications and enhancement of cancer patients’ quality of life is increasingly important.

Older patients also have multiple chronic morbidities. Primary care is well placed to address the sequelae of PCa and its treatment, alongside other long-term conditions [20, 21]. The provision of proactive care by general practice can increase the quality of integrated, efficient, and patient-centred care while reducing costs, and workload for specialists and improving continuity of care [20, 22–24]. PCa survivors also rated primary care clinicians significantly higher than oncologists in patient-centred cancer follow-up care [25]. The American Cancer Society (ACS) developed Prostate Cancer Survivorship Care Guidelines in 2014 to facilitate the provision of post-treatment care by primary care clinicians [21].

Implementation science is increasingly used to improve the implementation of evidence-based practice in health care [26]. It provides theoretical frameworks to gain insight into the mechanism by which implementation is more likely to succeed. The i-PARIHS framework (integrated-Promoting Action on Research Implementation in Health Services) has been widely used in health services to describe its dynamic and complex nature [27–29]. The core constructs of i-PARIHS are innovation, recipients, context, and facilitation [28]. The innovation construct focuses on sourcing and applying available research evidence whereby explicit knowledge is blended with tacit, practice-based knowledge. The recipient construct encompasses the people who are affected by and influence implementation. They have an impact on supporting, or resisting, innovation. The context construct consists of inner and outer contexts at the micro, meso, and macro levels (such as local, organisational, and external health system levels). It is defined in terms of resources, culture, leadership, and orientation to evaluation and learning. The facilitation construct is the process that activates implementation through assessing and responding to characteristics of the innovation and the recipients within their contextual setting. This requires a role (the facilitator) and a set of strategies and actions (the facilitation process) to enable implementation.

The aims of this review are to apply the i-PARIHS framework to synthesise evidence, analyse factors that influence the implementation of guideline-recommended care for maintaining bone health/fracture risk assessment, and identify strategies to improve bone health in this population. This will inform future research into the development and implementation of a complex intervention in primary care to reduce the risk of fractures in men with PCa-ADT.

## Methods

### Study design

A systematic scoping review methodology was selected to enable a broad review of the heterogeneous literature and identify knowledge gaps in the implementation of guidelines. The review was conducted according to the preferred reporting items for systematic review and meta-analyses extension for scoping reviews (PRISMA-ScR) guidelines [30].

### Inclusion/exclusion criteria

Studies that reported measures to improve fracture risk assessment for men with PCa-ADT in all healthcare settings were included worldwide. All study designs were included except descriptive articles, e.g., commentaries and editorials. The population was defined as patients with PCa taking ADT. The intervention criteria were studies designed to improve fracture risk assessment. Outcomes included: improvement in BMD measurement or fracture risk assessment, and/or changes in the prescription of a bone protective medication (BPM). Studies were excluded if: the research investigated the prevention of bone metastasis or skeletal-related events associated with metastatic bone diseases; were non-English; or only reported the efficacy of bone protective measures without intervention strategies.

### Search strategy

The search strategy was developed in consultation with information specialists at the University of Sheffield. Four databases (Medline, Embase, CINAHL, and Cochrane Library) were searched for studies published between 01/01/2000 and 18/01/2023. This data range was chosen to include contemporary findings since the recommendations were proposed. A supplementary search included searches on OpenGrey and Google Scholar as well as hand-searching for references and citation lists of the included articles. Search terms were MeSH terms and keywords related to prostatic neoplasms, androgen deprivation therapy, bone mineral density, fractures, and bone protective medicine (Supplementary Table 1).

### Reference screening

Search results were uploaded into Endnote (vX9.2) for screening. Two authors (QH, ET) independently reviewed the titles and abstracts of articles from the initial searches. Full texts of the articles were then retrieved for further assessment (QH, CM) for inclusion in the review. Disagreements were resolved through consensus (QH, CM, JB).

### Data extraction and synthesis

A data extraction spreadsheet was created and conducted by QH and checked for accuracy by CM. Both authors (QH, CM) discussed the extracted information with a third author (JB), deciding what information should be kept on the consensus. Key data included the year and country of the study, study design, purpose, participants, sample size, context, intervention/implementation strategies, and outcomes. A deductive approach for data synthesis was applied using the i-PARIHS framework. Data was evaluated under each construct of the framework to identify factors impacting the implementation. The sources of evidence were not appraised due to the nature of a scoping review according to PRISMA-ScR [30].

## Results

### Study selection

**Fig. 1:**
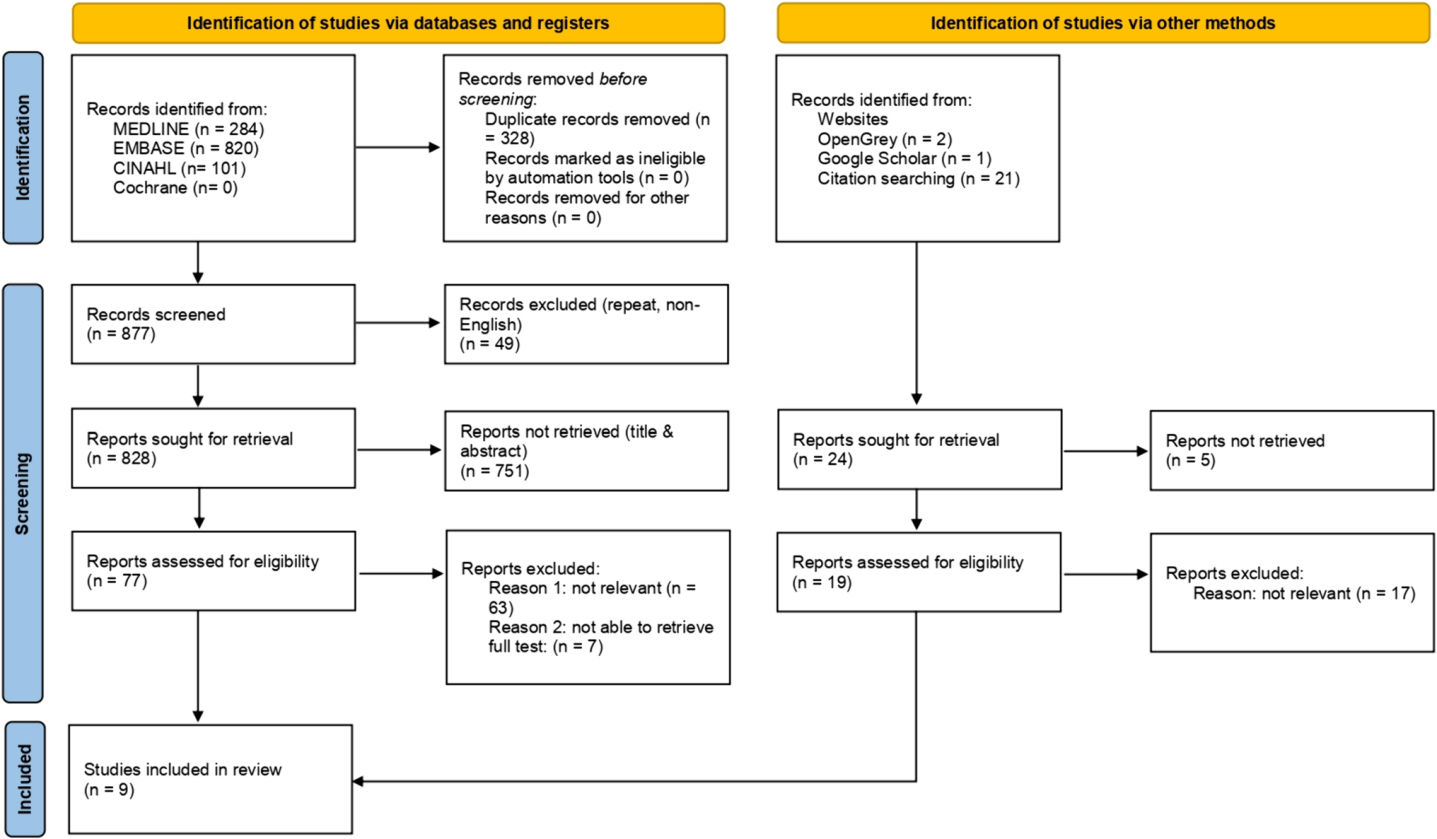
PRISMA flow chart

Our search protocol yielded a total of 1229 articles including 1205 from database search and 24 via other methods. After duplicates were removed, a total of 901 were screened by titles and abstracts, and 96 articles remained for full-text review for eligibility. A final count of 9 articles met all criteria and were included in this review. A PRISMA 2020 flow diagram illustrating the process of selecting articles is shown in Fig. 1.

### Characteristics of the included studies

Of the nine studies that were identified and met the criteria, six studies were conducted in Canada [31, 33, 35–38], one each in Australia [34], Belgium [32], and the US [39]. The study design consisted of one phase-2 randomised control trial (RCT) [31], three ‘before and after’ observational studies [32, 36, 38], one prospective cohort study [34], one retrospective observational study [33], one retrospective cohort study [39], and two surveys (clinicians [35] and patients [37]). Study data is summarised in Table 1 with more detailed information in Supplementary Table 2.

**Table 1:** A brief summary of the characteristics of the studies

Ref (author, year)	Place and study time	Objectives	Population (numbers, PCa status, ADT treatment)	Methodology	Intervention	Key findings
31 (Alibhai 2018)	**Canada** A tertiary care centre (Dec 2013–Nov 2014)	To assess 2 education-based models of care interventions to determine their feasibility and ability to improve bone healthcare	***N* = 112** (**40:36:36**) Men ≥ 50 Initiate or continue ADT for > 6 months Non-metastatic or castration-sensitive metastatic PCa	Phase 2, single-centre, parallel-group, 3-arm RCT (1:1:1), not blinded Data collected 6 months after randomisation	2 models of care were compared **1. Con** usual care **2. BHP + FP** (patient provided with **BHP** bone health pamphlet + brief recommendations for FP family physician) **3. BHP + BHCC** (bone health pamphlet for patients + support from **BHCC** bone health care coordinator)	**BMD test within 6 months** Con 36% BHP + FP 58% BHP + BHCC 78% **Bisphosphonate** was unable to be determined as no high fracture risk was detected hence no indication and prescription **Feasibility** Recruitment 68.4% Retention > 90% Satisfaction pt > 80% **Satisfaction FP 26%** Satisfaction specialist 80%
32 (Bultijnck 2018)	**Belgium** University hospital 2014 (before), 2015(after) Pathway was introduced in Jan 2015	To assess the effects of the implementation of a clinical pathway on evidence-based strategies for the management of ADT-induced side effects	***N* = 258 (before:after, 126:132)** All PCa patients require ADT > 6 months At the onset of ADT (within 3 months)	**Retrospective** **Before:after study** **1-year intervention**	Create an MDT for pathway development, implementation, and evaluation using a pathway framework The pathway consists of several risk screening assessments (bone, cardiac, metabolic) and preventative strategies Refer patients to a central pathway coordinator, who provides appointments	**Risk assessment before vs after** Bone (BMD/FRAX): 10% vs 58% Cardiac: 16% vs 61% Metabolic: 4% vs 46% **Advice for preventing strategies** Exercises 11% vs 62% Nutrition 10% vs 58% Psycho-education 13% vs 46% VitD Calcium 29% vs 67%
33 (Chahin 2016)	**Canada** A tertiary hospital (2010–2014)	To examine the quality of care provided to men on ADT who were seen in a specialised osteoporosis clinic, the compliance with guidelines including the use of validated fracture risk assessment tools, BMD request, and healthy bone lifestyle recommendation	***N* = 100** All stages of PCa Existing or newly starting ADT	**Chart review** 100 consecutive cases Data collection through review electronic records	Dedicated osteoporosis clinic, all patients were seen by one specialist specialised in male osteoporosis	**BMD** testing after ADT < 3 months: 40% 3 and 12 months: 17% > 1 year = 43%, of these 35% had first BMD in the clinic **Fracture risk assessment**—CAROC was used in all patients; 42 moderate risk, 12 high-risk **BPM**—All patients with high fracture risk were prescribed a bisphosphonate
34 (Cheung 2013)	**Australia** A tertiary teaching hospital (May 2007–July 2011)	To evaluate the effectiveness of implementing standardised guidelines to mitigate metabolic and bone side effects of ADT in men with non-metastatic PCa	***N* = 113** Patients with non-metastatic PCa started long-term ADT	**Prospective** cohort observational study, no control group 2-year follow-up	Refer to a dedicated MDT men’s health clinic Assessed and managed at 3–6 monthly intervals for bone and cardiovascular risk Also provided diet and lifestyle advice Overweight and obese men were offered a dietician referral BMD was measured at baseline and repeated annually	**BMD** *Baseline* Osteoporosis 23 (11%): **14 newly diagnosed** Osteopenia 86 (40%): **74 newly diagnosed** *At 2 years:* 84 had BMD tests If taking BPM, hip BMD maintained (0.885 vs 0.892 before vs after) If not taking BPM, BMD is reduced by 2.5% (1.021 vs 0.995) **BPM treatment** The number of patients who received BPM increased from 4 to 14
35 (Damji 2015)	**Canada** Across Canada (July–Dec 2012)	To determine PCa specialists’ knowledge, practices, self-perceived competencies, and barriers to providing guideline-concordant care in the diagnosis, prevention, and management of ADT-induced osteoporosis in PCa patients	***N* = 83** Practising urologists (recruitment 18.7%) ***N* = 73** Practising radiation oncologists (recruitment 60.8%)	**National Survey** Questionnaires were distributed both on paper and online Dillman’s tailored design method, 3-point contact to potential participants	Questionnaires assessing: 1. Knowledge 2. Self-assessed competencies 3. Current practices 4. Self-perceived barriers	Correctly identify the guideline-concordant DXA scans (76.3%), vitamin D (70.3%), and calcium (53.2%) intake and offer BPA treatment (57.6%) 32.5% measure BMD prior to ADT and 36.6% measure 1–2 years follow-up 4.6% used a validated fracture risk assessment tool 41% of urologists and 19% of radio oncologists would treat themselves for osteoporosis Competency in providing self-management education (40%) and managing osteopenia and osteoporosis (41%) The identified barriers were lack of time, structural support, training, and coordination among the healthcare team
36 (Jones 2022)	**Canada** A tertiary hospital The largest cancer centre in Canada The study date is not available	To implement and evaluate the impact of BoneRx on 1. Bone health care (BMD ordering, patient counselling); 2. Patient engagement in HBB, 3. Patient knowledge and health beliefs regarding osteoporosis 4. Patient satisfaction	***N* = 292** (before *n* = 143, after *n* = 149) PCa excludes chemotherapy or metastasis Understand English	Cross-sectional Before/after cohort study Patients’ questionnaire and chart review Follow-up at 6 months after ADT treatment	A prepopulated bone health prescription tool, entitled BoneRx, including BMD request and patient counselling of bone health, was provided at the initiation of ADT Patients were also provided with an educational booklet Multiple enabling and reinforcing strategies were used based on the Awareness-to-adherence and model of behaviour change	Before vs after BMD test: 34.7% vs 59.5% Patient bone health counselling: 32.4% vs 59.9% Vit D: 57% vs 81% Calcium supplement: 39% vs 61% Exercises: more engaged in moderate to vigorous activities No difference in patients’ osteoporosis knowledge, susceptibility, or seriousness Patient satisfaction: 7.8/10
37 (Nadler 2013)	**Canada** A tertiary hospital June–Dec 2011	To explore patients with PCa-ADT: 1. knowledge, self-efficacy (SE), and health beliefs about osteoporosis 2 current engagements in HBBs 3. the relationships between knowledge, SE, health beliefs, and engagement in HBBs	***N* = 175/330** (53% completion rate) Inclusion: current PCa-ADT, able to speak and read English Exclusion: concurrent chemo or had metastasis	**Questionnaire** Completed in the clinic or sent back by mail DXA requested if not received one in the past 18 months	Use the theory of Rosenstock’s Health Belief Model Questionnaires included 4 sections: 1. Demographic, 2. Osteoporosis risk factors, 3. HBB assessment, 4. Validated measures for knowledge, SE, health beliefs DXA was requested if not received in the past 18 months	**DXA scan in the past 2 years**: 38% **Osteopenia** (48%), **osteoporosis** (6%) **FRAX**: moderate risk 21%, high risk 2% **Osteoporosis knowledge**: low **Perceived SE**: moderate **Health motivation**: fairly high **Perceived susceptibility and seriousness of OP**: low Patients taking calcium, vitamin D and for exercise had significantly greater knowledge than those who did not
38 (Tsang 2018)	**Canada** Tertiary cancer centre Before: 2013–2014 After: (2014–2016)	To evaluate the ability of a multimodal patient education initiative to improve adherence to HBBs in men with PC-ADT	*N* = 51 before, (recruitment 86%) *N* = 52 after, (recruitment 72%) Received ADT < 12 months or plan to start ADT within 3 weeks for ADT more than 6 months Exclude: Unable to exercise, severe cardiac disease, stage IV V CKD	Pilot prospective, single-site, before-after clinical trial Patient self-report at regular 3 months follow-up	Multimodal HBB education: BoneRx (including DXA refer) + Focused F2F education with an oncology nurse 60% or physician 40% Patients were provided with customised educational materials	Patients were satisfied with the study intervention, found educational materials easy to understand, and felt that it increased their knowledge about osteoporosis The intervention appeared to be associated with trends toward improved HBB; none of them were statistically significant. Changing patient behaviours may require more than a brief one-time educational intervention to be effective Patients who received the study intervention were more likely to receive BMD testing (OR 3.3, 95%CI 1.3–8.8)
39 (Zhumkhawala 2013)	**US** Within a primary care population-based membership programme 2003–2009	To determine whether the implementation of the HBP (healthy Bone Program) screening and treatment protocol was effective in reducing the rate of osteoporotic hip fracture in men with PCa-ADT	***N* = 1482** 1071 intervention, 411 control Newly diagnosed PCa received leuprolide as monotherapy, no previous hip fractures Exclude pathologic/traumatic hip fracture	**Retrospective** cohort Electronic medical record systems and cancer registries were the primary data source	**Intervention**: through an existing osteoporosis prevention program **Control**: all others not in the programme	**Incidence of hip fracture per 1000-person year** Reduced by 70%. Intervention 5.1 (3.0–8.0) vs Con 18.1 (10.5–29) **Hip fracture mean interval between event date and first ADT injection** Delayed in the Intervention group 828.7 days vs Con 590.3 days **Receive BPM treatment** Intervention 29.2% vs Con 3.2%

### The i-PARIHS framework analysis

Table 2 summarises the findings using the iPARIHS framework with references to the studies aligned with each construct.

**Table 2:** Summary of the key findings using the i-PARIHS framework

Innovation	Context	Recipients	Facilitator/facilitation
**Source of innovation** Starting point: evidence-based national guidelines and recommendations are viewed as robust. Clinical practice indicates poor compliance and insufficient bone care health for ADT users in all studies [31–39] Theories: a pathway framework for pathway development, implementation, and evaluation [32], a health belief model to support education impact on uptaking bone health care guidance [31, 36, 38] Experiences: from evidence in women with osteoporosis [31] Literatures: Point-of-care reminders increased adherence to clinical recommendations [36] **Strategies** Using an existing bone health care programme to proactively screen and treat patients Proactively target men with PCa receiving long-term ADT within an existing generic osteoporosis prevention programme [39] Development of new services with new care pathways or clinics MDT (8 disciplines) was created for pathway development, implementation, and evaluation according to a seven-phase pathway framework [32]. The MDT analysed daily practices, deficits in the care process (e.g., lack of time and resources), and transferrable components of pathways. In addition to bone health, the pathway also assessed cardiometabolic side effects and provided advice for exercises, nutrition, and psychoeducation [32] Referred patients to a dedicated men’s health clinic where metabolic and bone side effects were assessed and managed [34] Assessed in an osteoporosis clinic by one doctor who specialised in male osteoporosis with experience in ADT treatment [33] Incorporating BMD tests into routine patient care A pre-populated prescription including a BMD request was provided to the PCa specialists to prompt them to request BMD tests [36, 38] BMD tests were requested by family physicians or a bone care coordinator [31]	At the organizational level, either within a hospital or a community health care provider, all studies were driven for change by the gap between guidelines and poor practice [31–39] High research activities within the largest cancer centre indicate a high level of support and leadership within the organisation [31, 36–38]	**Clinicians** Urologists and oncologists reported lack of time, resources, and supporting structure were the most significant barriers [32, 35] Lack of training in assessing fracture risk assessment, and confidence in providing advice to patients regarding bone health self-management [35] Lack of awareness of ADT-induced bone loss and the perception that this will be looked after by the patient’s primary care clinicians [33] Challenges in team working, family physicians reported a low satisfaction rate when bone care was transferred to them from the hospital [31]. Hospital letters were not timely, clearly written, or helpful [31] **Patients** As part of the stakeholders who give feedback on intervention materials and workflow to fine-tune the implementation approach [36] Lack of basic information about ADT-induced bone complications despite their relatively high health motivation [37] Received information and education on bone health and ADT complications [31, 36, 38]	Poor reporting of facilitators and facilitation process, and lack of details in evaluation and adaption in most of the studies, hinders the identification of the critical component of the success of an intervention Care coordinators to facilitate new services [31,32], no detailed information of their speciality background Based on the Awareness-to-Adherence mode of behaviour change [36] Promote awareness and agreement: gather feedback from stakeholders (HCPs and patients) and present to the site teams at weekly rounds Following initial implementation (3 months), facilitate adoption and adherence: audit and feedback, reminders were integrated into routine clinical care team meetings, developed information posters as reminders [36] Increase staff capacity to shorten the referral waiting time at the start of a new osteoporosis clinic [33] Active screening and treatment protocols are easily implemented in an established healthy bone programme using automated systems and a comprehensive electronic medical record [39] Lack of evaluation Lack of long-term strategies for sustainable services [31, 38]

### The innovation construct

#### Source of innovation (underlying knowledge)

All studies were based on published recommendations to reduce osteoporosis and fracture risk for PCa-ADT users. A health belief model was used by a Canadian research team to support the importance of patient education in delivering care [31, 36, 38]. They also adopted their intervention approach using strategies for women with osteoporosis [31], and findings from a previous systematic review of point-of-care reminders to increase adherence to recommendations [36]. A pathway framework was applied when a new pathway was developed by a Belgium team in referring patients for the comprehensive management of ADT-induced complications [32].

### Types of innovation

#### Using an existing bone health care programme to proactively screen and treat patients

A study in the US proactively identified PCa-ADT patients within an existing osteoporosis prevention programme for members of a healthcare community [39]. Using the electronic medical record system and cancer registry, the authors found screening and treatment protocols were easily implemented. The patient population in the study was also large and diverse and had equal access to healthcare (The study contained 1482 PCa-ADT patients including 17% black men). The study reported a 70% reduction in hip fractures (from 18.1 to 5.1 per 1000 person-years) and an increased uptake of BPM (from 3.2% to 29.2%). For patients who sustained a hip fracture, the median interval from the first ADT treatment to hip fracture was also longer in the intervention group (801 vs 528 days) [39].

#### Development of new services with new care pathways or clinics incorporating existing services

New pathways or clinics were developed in three studies, all at a tertiary hospital level [32–34]. In a before–after study in Belgium, a multidisciplinary team consisting of a radiation oncologist, urologist, psychologist, dietician, oncology nurse, physical therapist, social worker, and pathway facilitator was created for pathway development, implementation, and evaluation according to a seven-phase pathway framework [32]. The multidisciplinary team analysed daily practices, deficits in the care process, and potential transferrable components of pathways. In addition to bone health, the pathway also assessed cardiometabolic side effects and provided advice for exercises, nutrition, and psychoeducation to patients. BMD screening increased from 10 to 58% after the pathway implementation [32]. The positive effects are attributable to standardisation of the care process [32].

Patients were also referred to a new dedicated clinic, for example, a Men’s Health Clinic by an Australian team where metabolic and bone complications were assessed and managed. After 2 years of attendance at the clinic, the proportion of men receiving BPM increased from 17 to 61%. Treatment maintained total hip BMD (+ 0.007 ± 0.239 g/cm2, *p* = 0.649) while a significant decline in hip BMD by 2.5% (− 0.026 ± 0.036 g/cm2, *p* < 0.0001) was reported in those without treatment [34].

A Canadian team referred patients to a bespoke osteoporosis clinic where they were seen by one doctor who specialised in ADT treatment-induced osteoporosis [33]. High-quality bone health care was achieved as defined by a valid fracture risk assessment tool used in all patients, and all patients with high fracture risk were recommended BPM [33].

#### Incorporating BMD tests into routine patient care

Researchers from Canada developed different ways to implement BMD tests for PCa-ADT patients. A healthy bone prescription tool entitled BoneRx that contained a pre-populated BMD request was provided to the PCa specialists to prompt them to order a BMD test for PCa-ADT patients. In a pilot prospective, before-and-after clinical trial, at 3-month follow-up, patients who received the study intervention were more likely to receive BMD testing (odds ratio 3.3) compared to the control group [38]. In a recent implementation study, BoneRx was provided to patients at the initiation of ADT. After 6 months of ADT treatment, significantly more patients received BMD tests (59.5%) compared with patients who did not receive BoneRx (34.7%) [36]. In another study, two strategies of requesting BMD tests were compared: BMD was ordered either by the patient’s family physicians or facilitated by a bone healthcare coordinator. Patients were also provided with a bone health pamphlet. Significant increases in receiving BMD tests within 6 months were seen in both groups (58% with family physicians, 78% with a coordinator) as compared with the usual care group (36%) [31].

### The context construct

At an organisational level, either within a hospital or a community health care provider, all studies were driven by the gap between guidelines and poor real-world practice [31–39]. However, there was no direct information reported in any study related to local settings that influenced the implementation. A research group from the largest cancer centre in Canada conducted a number of research activities to investigate ADT-induced bone complications, indicating a high level of support and leadership within the organisation [31, 36–38]. No studies reported any information related to external contextual factors, e.g. policy or economic drivers.

### The recipients construct

#### Skills, knowledge, and resources for clinicians

Urologists and oncologists should prescribe ADT with the knowledge of its complications on bone loss [33]. However, they reported lack of time, supporting structure, and resources as major barriers [32, 35]. To address this, a multidisciplinary team developed a new pathway that incorporated transferrable existing services. A central pathway coordinator was created to facilitate the referral [32]. Lack of training and confidence were also reported in a survey from Canadian radiation oncologists and urologists [35]. Only 4.6% of survey respondents routinely used fracture risk assessment, and 37.3% had never even heard of them [35]. When osteoporosis was detected in PCa-ADT patients, referrals were commonly made to the patient’s primary care physician (47.4%), endocrinologist (23.1%), or an osteoporosis clinic (19.2%) [35]. Chahin et al. [33] also reported major barriers to optimising bone health for men on ADT are the lack of knowledge among PCa specialists and the perception that this issue would be looked after by the patient’s primary care clinicians [33]. It was suggested that referring patients to primary care clinicians or bone health specialists might be appropriate [33]. The research team subsequently conducted studies that involved family physicians or osteoporosis specialists’ referral. In a randomised phase 2 clinical trial to assess two education-based interventions to improve bone health care, one strategy was to refer patients to their family physicians who were provided with a hospital letter that contained recommendations for BMD screening and bone health care information [31]. The result showed that with the family physician’s input, BMD ordering was significantly increased compared with usual care (58% vs 36%, *p* = *0.047*). In another study, patients were referred to a dedicated osteoporosis clinic which was specialised in ADT-induced bone loss. Patients received high-quality bone healthcare: all patients received fracture risk assessment and BPM was offered to all patients with a high risk of osteoporosis [33].

#### Support and collaboration at a multidisciplinary team level

The novel patient care pathways were predominantly multidisciplinary [31, 32, 34, 36, 38, 39]. However, perspectives from different specialists on the new services were usually not reported. In the above phase 2 clinical trial involving family physicians, despite an improvement in bone health care, family physicians reported a very low satisfaction rate (26%), which was the only feasible outcome that did not meet the target [31]. Problems included hospital letters that were neither timely, clearly written nor helpful. The authors recommended eliciting feedback from family physicians before embarking on a larger trial [31].

#### Patients as recipient

Patients as service users fit into a wide range of stakeholders within the recipient construct, and their views should be consulted [28]. Only one study described gathering feedback from patients as well as clinicians on intervention materials and workflow to fine-tune the implementation approach [36]. However, no detailed information, such as what patients’ input was or whether any changes were made from patients’ feedback, was reported [36].

A survey with 175 PCa-ADT patients in Canada exploring their knowledge and healthy bone behaviour (HBB, including calcium and vitamin D intake and exercises) showed that most patients lacked basic information and awareness of ADT-induced bone complications despite their relatively high health motivation [37]. The findings supported the application of the Health Belief Model in this population that increasing knowledge of bone health may increase compliance with HBB guidelines, and highlighted the importance of patient education [37]. The research team then incorporated patient education together with a healthy bone prescription tool, i.e. BoneRx, for the clinicians to prompt them to request BMD tests [31, 36, 38]. Different strategies were developed to provide information and education to patients. For example, a customised written booklet on bone health was created and given to patients at the initiation of ADT [36] or during ADT treatment [31, 38]. The booklet was created through a collaboration with specialists in osteoporosis and PCa, and the hospital patient education department [31, 36, 38]. Patients also received counselling, either face-to-face with specialists [36, 38] or from a bone health care coordinator [31]. BMD tests were improved in all three studies (from just over 30% to more than 50%) [31, 36, 38]. Interestingly, no differences were detected in patients’ osteoporosis knowledge or feelings of osteoporosis susceptibility, or osteoporosis seriousness [36, 38]. It was suggested that providing patients personalised DXA results or risk information [38] as well as more than a brief single educational intervention may be effective [36, 38]. The authors also suggested reinforcing change over time by use of repeated follow-up encounters, and the need to explore and address patient barriers and facilitators of lifestyle change [38].

### Facilitator and facilitation construct

#### Poor reporting of facilitator processes

There is a lack of detailed information about the facilitation process in most of the studies [31–34, 38, 39]. This hinders the identification of the critical components of the success of an intervention.

#### Apply a care coordinator to facilitate the new services

A bone health care coordinator and a central pathway facilitator were reported in 2 studies respectively [31, 32]. Applying evidence from studies in women with osteoporosis, a strategy of having a patient bone health care coordinator was used in a study comparing two education-based models of care study [31]. The role of a care coordinator was to go through the educational material with the patient using principles of adult education and chronic disease self-management. They also followed up with the patient at least twice over 3 months to facilitate behavioural change and BMD ordering [31]. The authors reported a great likelihood of undergoing a BMD test (odds ratio 8) if patients were assigned to the co-ordinator group when compared with usual care [31]. In the pathway study managing multiple ADT-induced side effects, the coordinator received referrals of patients from radiation oncologists and urologists. They discussed the pathway with the patients and provided follow-up appointments for screening assessments and preventative strategies including fracture risk assessment [32]. The referral rate was reportedly suboptimal (61%) in the first year that the pathway was implemented but was expected to further increase [32]. No information was given regarding the speciality background of the facilitators in either study, i.e., whether the person was a nurse, administrator, or clinician and from which medical speciality [31, 32].

#### Applying a theory-based implementation strategy to facilitate the intervention

A theory-based implementation strategy was used to facilitate the intervention with a new healthy bone prescription tool [36]. Based on the Awareness-to-Adherence mode of behaviour change, the authors applied multiple enabling and reinforcing interventions. Strategies included presentations to the site teams at weekly tumour rounds to promote awareness and agreement, and audit and information posters to facilitate adoption and adherence [36]. In the above pathway study, the new pathway protocol as well as an implementation plan were developed according to a pathway framework. However, no information was reported about how the pathway was implemented or facilitated [32].

#### Evaluation

Evaluation was often not included or reported. In a phase 2 clinical trial assessing an education-based intervention, the authors investigated the feasibility of the study and found good recruitment (exceeded the target of 60%), retention (over 90%), patient and specialist satisfaction (over 90%), and outcome capture (over 90%), although the satisfaction rate for the family physician was low (26%) [31]. Evaluation was also included in the development of a new multidisciplinary pathway service using a pathway development framework [32]. However, no information about the evaluation was reported [32].

Concerns about cost-effectiveness were raised in two studies that involved specialists providing patients’ education and BMD requests [31, 38]. It was suggested that referral to family physicians was less resource-intensive [31], while oncologist delivery of the intervention could adversely affect clinic flow [32]. Most studies were performed in a tertiary hospital with data collected from 6 months to 6 years. Ongoing re-evaluation of the care pathway and strategies to ensure that the new service is sustainable are all required if implementation is to be applied to wider settings and a longer term.

## Discussion

This scoping review analyses factors that influence the implementation of the guidelines for reducing fracture risk for patients with PCa receiving ADT. An i-PARIHS implementation framework was used to synthesise the data. Under the innovation construct, several strategies were reported such as developing a new care pathway using a multidisciplinary approach [32], using an existing bone healthcare programme to proactively screen and treat patients [39], developing dedicated bone health clinics for ADT users [33, 34], and providing a pre-populated prescription tool for clinicians to prompt BMD requests [36, 38]. Under the recipient construct, we identified barriers to the implementation including lack of awareness of the ADT-induced bone complications for both clinicians [32, 35] and patients [36–38], lack of time, resources, and structure for clinicians in providing bone care for the patients [32, 35], and lack of communication between specialists and family physicians [31]. A complex intervention that included the provision of information and education for patients improved the uptake of BMD requests [31, 36, 38]. However, there was a lack of detailed information in the context and facilitation constructs in most of the studies, which limits the identification of key elements for a successful implementation.

Studies in older adults have shown that providing education and counselling to patients is effective in improving patients’ knowledge of osteoporosis, increasing calcium and vitamin D intake, increasing exercise, and an improvement in BMD tests [40]. Research in postmenopausal women [41] and women with breast cancer taking hormone deprivation therapy [42] has also shown that education has a positive impact on improving bone health. It is interesting that our review found that providing information and education to PCa-ADT patients did not increase their osteoporosis knowledge, or feelings of osteoporosis susceptibility or seriousness [36, 38]. The reasons that patients seemed not to be getting the message may be that they did not read the information, or did not understand or remember what they were being told [36], and the fact that the focus of these appointments was primarily the controlling of malignancy [35]. The management of bone health complications (which can remain asymptomatic unless a fracture occurs) may not be at the forefront of patients’ priorities [43]. In a national survey in the US among physicians in Primary Care, Endocrinology, and Geriatrics, the most commonly reported barrier to osteoporosis screening was patient non-adherence [44]. Patients as service users fit into a wide range of stakeholders within the recipient construct of the iPARIHS implementation framework [28]. However, their views were not often consulted during intervention development. Future studies need to explore and address barriers for PCa-ADT patients to receive education and comply with fracture risk assessment and treatment [38]. The development of new care services requires patients’ participation to tailor the interventions to their needs.

Although the aim of our study was to collect data from all healthcare settings, most studies were in tertiary hospitals. This is possibly because ADT is usually initiated by urologists or oncologists and most guidelines recommend screening for osteoporosis with BMD testing before or at the start of ADT [13–15]. Our study identified the lack of time, knowledge, and supportive structures for PCa specialists to implement evidence-based guidelines in mitigating bone complications from ADT treatment [32, 35]. One possible strategy is to provide a pathway together with an implementation plan [32]. Clinical pathways are often used to optimise adherence to guidelines [45]. They are widely used to structure and standardise evidence-based care processes and improve the quality of care and patient outcomes [46]. PCa specialists could also be prompted to order a BMD test by providing them with a pre-populated healthy bone prescription tool [36, 38]. Incorporating BMD measurement into routine patients’ care effectively facilitated the uptake of fracture risk assessment for this population.

Two studies reported referring patients to a care co-ordinator to facilitate the new services [31, 32]. Applying facilitator roles to support the implementation of changes is common in healthcare practice [28]. Facilitators can be internal or external or a combination of the implementation setting [28]. However, the speciality background of the facilitators is not clear in either study [31, 32]. In addition, there is also a lack of reporting facilitation or evaluation in most of the studies. This has hindered the identification of the critical factors that have impacted the success of an intervention.

Only two studies involved community healthcare providers [31, 39]. As PCa survival rate improves, primary care physicians could play a larger role in managing cancer treatment-induced long-term complications. Involving primary care physicians in bone health for PCa-ADT patients can result in an improvement in BMD requests [31]. Our review reported that there was a lack of knowledge and training in bone health among PCa specialists [33, 35], and the perception that this aspect of care would be addressed by the patient’s primary care clinicians [33]. Almost half of oncology radiologists and urologist specialists would make referrals to primary care physicians when osteoporosis was identified [35]. Primary care physicians also provide continuous care and often administer ADT injections after ADT is initiated by the specialists. Therefore, there is an opportunity to follow-up PCa-ADT patients and offer information about bone health. This is important as our review suggested that one-off motivation and education by a specialist do not support patients to sustain healthy behaviours to maintain bone health in the long term [36, 38].

However, our review has identified that the primary care clinicians reported poor satisfaction despite improved patient care in increased BMD requests [31]. Problems included hospital letters not being timely, clearly written, or helpful [31]. This highlights the challenges in shared care for cancer patients between specialists and primary care. Poor communication of follow-up plans from specialists as well as lack of knowledge in cancer care for primary care clinicians has been frequently documented [20, 23, 24]. It was reported in the UK that less than half of GPs considered a previous history of cancer or cancer treatment when assessing bone or cardiovascular health [24]. In a recent scoping review of barriers and solutions to the implementation of primary care provider-led cancer survivorship care, potential solutions were proposed including improving interdisciplinary communications, bolstering education, and providing survivorship resources [23].

In the UK, cancer specialists usually provide a patient’s cancer summary to primary care with information including the patient’s cancer diagnosis, treatment, and monitoring advice. GPs usually review patients at 3 months and 12 months after they are diagnosed with cancer, often with templates for review. It is possible to incorporate fracture risk assessment / BMD tests during the routine cancer review. Our study suggests that using a pre-populated prescription tool helped to prompt clinicians to request BMD tests [36, 38]. A structured cancer review using a template including fracture risk assessment could potentially improve the quality of bone health care for PCa-ADT patients delivered by primary care clinicians. Future study requires the co-design of care pathways by specialists, primary care clinicians, and patients.

## Strengths and limitations

To our knowledge, this is the first time that an implementation framework has been used to analyse the implementation of guidelines to mitigate the bone complications of ADT for patients with PCa. It is rare to take evidence in the original form of clinical guidelines and directly apply it within an implementation project [28]. Explicit knowledge is usually blended with practice-based knowledge and adapted to suit a particular situation [28]. The advantage of using a theoretically informed i-PARIHS implementation framework is to gain insights into the mechanism and implementation strategies. This evidence synthesis could inform service redesign in different settings including primary care.

There are some limitations of the study. The majority of the studies were from Canada. Most studies also lacked detailed information on how the implementation was conducted. It is difficult to identify the key elements that influence the uptake of the guideline in practice. The low number of studies included in the review indicated a lack of research evidence in this area, and hence an urgent need for more research.

## Implications for research and/or practice

Around 490,000 men are living with and after PCa in the UK [2] and at least a third require ADT in their cancer treatment journey [4]. There is consequently a significant risk of treatment-induced bone complications, and hence urgent action is needed to mitigate this risk by bone health for patients taking long-term ADT. Our review suggests a multidisciplinary approach can be effective, however, better communication, including communications between specialists and primary care physicians, should be explored in future research. In addition, we propose primary care could have a larger role in the management of bone complications for long-term ADT users, especially if the practice offers ADT administration or a cancer care review. Patients, families, specialists, and primary care clinicians should all be consulted in service development and participate in the co-design of interventions. Economic evaluation of innovative services should also be undertaken, considering the patient and healthcare burden of fractures.

## Conclusions

There is a need to strengthen evidence-based bone health management for PCa survivors taking ADT. This study has highlighted some barriers and potential strategies to increase the uptake of fracture risk assessment using the i-PARIHS implementation framework. Due to the multidisciplinary team involvement in bone care, a structured service should incorporate different disciplines with good communication. Patient education can also be beneficial, and their perspectives on bone health need to be further explored and addressed to enable more personalised education for patients.

## Supplementary Information

Below is the link to the electronic supplementary material.

Supplementary file1 (DOCX 12 KB)

Supplementary file2 (DOCX 33 KB)

## Data Availability

The dataset generated and analyzed during the current study are available from the corresponding author on reasonable request.
