# Supplementary material for: Implementation of fracture risk assessment in men with prostate cancer requiring long-term androgen deprivation therapy: a systematic scoping review using the i-PARIHS implementation framework
Source: J Cancer Surviv. 2024 Aug 14;20(2):399–414. doi: 10.1007/s11764-024-01659-3 (PMC12988880; doi:10.1007/s11764-024-01659-3)
Supplement: Supplementary file 1 — Supplementary file1 (DOCX 12 KB) [file 11764_2024_1659_MOESM1_ESM.docx]

**Supplement Table 1: Ovid MEDLINE search strategy**

Ovid MEDLINE Jan 2000 – Jan 2023

1. Prostatic Neoplasms/ 144161

2. (prostat$ adj3 (malignant or cancer or carcinoma or adenocarcinoma or tumo*)).mp. [mp=title,

book title, abstract, original title, name of substance word, subject heading word, floating sub-

heading word, keyword heading word, organism supplementary concept word, protocol

supplementary concept word, rare disease supplementary concept word, unique identifier,

synonyms, population supplementary concept word, anatomy supplementary concept word] 170603

3. 1 or 2 195323

4. Androgen Antagonists/ 13353

5. Gonadotropin-Releasing Hormone/ 29694

6. (ADT or androgen deprivation therapy or LHRH or GnRH or Buserelin or Goserelin or Leuprorelin

or Triptorelin or Leuprolide or Degarelix or Relugolix or hormon*).mp. [mp=title, book title, abstract,

original title, name of substance word, subject heading word, floating sub-heading word, keyword

heading word, organism supplementary concept word, protocol supplementary concept word, rare

disease supplementary concept word, unique identifier, synonyms, population supplementary

concept word, anatomy supplementary concept word] 854581

7. 4 or 5 or 6 859243

8. Accidental Falls/ 28135

9. Osteoporotic Fractures/ 8086

10. (fall* or fracture* or bone mineral density or BMD or DXA or DEXA).mp. [mp=title, book title,

abstract, original title, name of substance word, subject heading word, floating sub-heading word,

keyword heading word, organism supplementary concept word, protocol supplementary concept

word, rare disease supplementary concept word, unique identifier, synonyms, population

supplementary concept word, anatomy supplementary concept word] 675073

11. 8 or 9 or 10 675073

12. Diphosphonates/ 17533

13. (bisphosphonate* or alendron$ or zolendron$ or risedron$ or ibandro* denosumab).mp.

[mp=title, book title, abstract, original title, name of substance word, subject heading word, floating

sub-heading word, keyword heading word, organism supplementary concept word, protocol

supplementary concept word, rare disease supplementary concept word, unique identifier,

synonyms, population supplementary concept word, anatomy supplementary concept word]

24318

14. 12 or 13 30855

15. 3 and 7 and 11 and 14 290

16. limit 15 to yr=&quot;2000 - 2022&quot; 284
