# Supplementary material for: Implementation of fracture risk assessment in men with prostate cancer requiring long-term androgen deprivation therapy: a systematic scoping review using the i-PARIHS implementation framework
Source: J Cancer Surviv. 2024 Aug 14;20(2):399–414. doi: 10.1007/s11764-024-01659-3 (PMC12988880; doi:10.1007/s11764-024-01659-3)
Supplement: Supplementary file 2 — Supplementary file2 (DOCX 33 KB) [file 11764_2024_1659_MOESM2_ESM.docx]

**Supplement Table 2 Characteristics of the studies**

| **Ref,**  **Author**  **year** | **Place and study time** | **Objectives** | **Population** | **Methodology** | **Intervention** | **Outcome** | **Conclusion** |
| --- | --- | --- | --- | --- | --- | --- | --- |
| **31**  **Alibhai**  **2018** | **Canada**  A tertiary care centre  Dec 2013 – Nov 2014 | To assess 2 education-based models of care interventions to determine their feasibility and ability to improve bone health care | **N= 112** (**40:36:36**)  (con:BHP+FP:BNP+BHCC)  Inclusion criteria:  Men>=50  Initiate or continue ADT for > 6 months  Non metastatic or castration sensitive metastatic PCa  Life expectancy > 6 months  No BMD test or osteoporosis clinic visit < 2 years  Fluency in English | Phase 2, single-centre, parallel-group, 3-arm RCT (1:1:1), not blinded  Data collected 6 months after randomisation | 2 models of care  **1. Con** Usual Care  **2. BHP + FP** (patients provided with **BHP** bone health pamphlet + brief recommendations for **FP** family physician including BMD request)  **3. BHP + BHCC** (bone health pamphlet for patients + support from BHCC  BHCC went through educational material, follow up at least twice over 3 months | **BMD test**  Con 36%  BHP+FP 58%  BHP + BHCC 78%  **Appropriate Calcium intake** (compared with Con)  BHP+FP 15% greater  BHP + BHCC 5.6% greater  **Vit D** no changes  **Lifestyle** no changes  **Bisphosphonate** unable to determine as no high fracture risk was detected  **Feasibility**  Recruitment 68.4%  Retention >90%  Satisfaction pt >80%  **Satisfaction FP 26%**  Satisfaction specialist 80% | Educational strategies to improve bone health care appear feasible and are associated with improved BMD ordering in men receiving ADT  Low satisfaction with FP. FP feedback will be required to ensure letter clear and helpful, be received in a timely manner.  Require a formal cost-effectiveness analysis in future study |
| **32**  **Bultijnck**  **2018** | **Belgium**  University hospital  2014 (before) 2015(after)  Pathway was introduced in Jan 2015 | To assess the effects of a PCa pathway on the implementation of evidence-based strategies for the management of ADT induced side effects | **N= 258 (before:after)**  **(126: 132)**  Inclusion:  All PCa patients receiving ADT > 6 months, including metastasis  Exclusion: Karnofsky performance score <=50 | **Retrospective**  **Before: after study**  1 year intervention | Create an MDT (radiation oncologist, urologist, psychologist, dietician, oncology nurse, physical therapist, social worker and pathway facilitator) for pathway development, implementation and evaluation using a pathway framework.  The pathway consists of several risk screening assessments (bone, cardiac, metabolic) and preventative strategies (a supervised exercises programme, dietician referral, appointment with a psychologist, and advice on vit D and calcium.  Radiation oncologists and urologists refer the patients to the pathway coordinator, who provided f/u appointments for the screening assessments and preventive strategies.  The pathway undergoes a continuous evaluation process at both patient and healthcare professional level. | **Risk assessment before vs after**  Bone (BMD/FRAX): 10% vs 58%  Cardiac: 16% vs 61%  Metabolic: 4% vs 46%  **Advice for preventing strategies**  Exercises 11% vs 62%  Nutrition 10% vs 58%  Psycho-education 13% vs 46%  VitD Calcium 29% vs 67%  In the intervention group  BMD result: Normal 58.9%, Osteopenia 28.8%, Osteoporosis 12.3% | A clinical pathway improved the implementation of evidence-based strategies for the management of ADT-induced side effects  A clinical pathway could serve as a method to bridge the gap between evidence-based guidelines and daily clinical practice.  61% referral rate didn’t meet implementation level 80-90%, but is expected to increase.  Critical components of the success of the pathway need to be examined. The pathway needs to be tested in different hospitals.  Assess the long-term application of the pathway. |
| **33**  **Chahin**  **2016** | **Canada**  A tertiary hospital  2010-2014 | To examine the quality of care provided to men on ADT who were seen in a specialised osteoporosis clinic: the compliance with guidelines including use of validated fracture risk assessment tools such as FRAX or CAROC, BMD request, and healthy bone lifestyle recommendation | **N=100**  All stage of PCa  existing or newly starting ADT  ADT indication:  Primary (3 %), adjuvant (37 %), biochemical relapse (54 %), and metastatic disease (6 %)  Average ADT 21.4 (0.5-175) months at the time of baseline visit | **Chart review**  (100 consecutive cases)  Data collection through review electronic records | Dedicated osteoporosis clinic, all patients were seen by one specialist specialised in male osteoporosis | **Time of referral after ADT initiation**  < 3 months: 8%  3-12 months: 23%  > 1 year: 68%  **BMD** testing after ADT  < 3 months: 40%  3 and 12 months: 17%  >1 year = 43%, of these 35% had first BMD in the clinic  **Fracture risk assessment** - CAROC was used in all patients; 42 had a moderate 10-year fracture risk and 12 were high risk  **BPM** - All patients at high fracture risk were prescribed a bisphosphonate.  **Lifestyle recommendation**  Alcohol: 33% (2/6 patients)  Smoking: 80% (4/5 patients)  Exercises: 73% (16/22 patients) | The osteoporosis clinic performed a comprehensive assessment and recommended guideline-based bone health care for most men on ADT, suggesting a systemic approach, e.g. referring to a specialized osteoporosis clinic, to assessing bone health is associated with high rates of guideline-adherent care.  No referral details and rate were reported. |
| **34**  **Cheung**  **2013** | **Australia**  A tertiary teaching hospital  May 2007 – July 2011 | To evaluate the effectiveness of implementing standardised guidelines to mitigate metabolic and bone side effects of ADT in men with non-metastatic PCa | **113** available data (Baseline N=236  2 years f/u N=153)  Inclusion:  All patients with non-metastatic PCa started long-term ADT | **Prospective** cohort observational study, no control group  2 year follow up | Refer to a dedicated MDT men’s health clinic.  Assessed and managed at 3-6 monthly intervals for bone and cardiovascular risk.  Also provided diet and lifestyle advice.  Overweight and obese men were offered a dietician referral.  BMD was measured at baseline and repeated annually. | **BMD**  *Baseline*  Osteoporosis 23 (11%): **14 newly diagnosed**  Osteopenia 86 (40%) – **74 newly diagnosed**  *At 2 years:* 84 had BMD  If with BPA, BMD maintained (0.885 vs 0.892 before vs after)  If without BPA, BMD reduced by 2.5% (1.021 vs 0.995)  **BPM treatment**  Number of patients received BPM increased from 4 to 14  **Metabolic**  *Baseline:*  87% overweight or obese  61% hypertension  56% hypercholesterolaemia  27% CVD  Anaemia 13.8%  *2 years:*  Increase in waist by 2.8cm, HbA1C by 0.13% in men without diabetes  Anaemia 32.5%  Reduction in cholesterol -0.35, BP -7.6/-4.7 mmHg. | Findings indicate that a structured approach effectively prevents bone decay over 2 years.  Larger studies are needed to determine effects on preventing fractures, cardiovascular events and death.  Cost-effective model of care will require future study. |
| **35**  **Damji**  **2015** | **Canada**  Across Canada  July – Dec 2012 | To determine PCa specialists’ knowledge, practices, self-perceived competencies and barriers to providing guideline-concordant care in the diagnosis, prevention, and management of ADT-induced osteoporosis in PCa patients | **N=83**  Practising urologists (recruitment 18.7%)  **N=73**  Practising  radiation oncologists  (recruitment 60.8% | **National Survey**  Questionnaires distributed both on paper and online  Dillman’s tailored design method, 3-point contact to potential participants | Questionnaire consists of:  (i) knowledge regarding osteoporosis and guidelines for bone health management in men receiving ADT,  (ii) self-assessed competencies regarding bone health management,  (iii) current practices regarding osteoporosis prevention and management,  (iv) self-perceived enablers and barriers to the uptake of the current guidelines, | The majority of respondents were able to correctly identify the guideline-concordant frequency of repeat DXA scans (76.3 %), vitamin D (70.3 %), and calcium (53.2 %) intake and offer BPA treatment (57.6 %).  32.5 % reported routinely measuring BMD prior to starting ADT and 1–2 years following the initiation of ADT (36.6 %).  Only 4.6% routinely used a validated fracture risk assessment tool.  When osteoporosis was detected, 41% urologist 19% radio oncologists would treat themselves. Referral were made to primary care physician 47%, endocrinologist 23%, or an osteoporosis clinic 19%.  Self-assessed competency in providing self-management education to patients (40%) and managing patients with osteopenia and osteoporosis (41%).  The identified barriers were lack of time, structural support, training, and coordination among the healthcare team. | Despite high knowledge about appropriate bone health care among PCa specialists, there remain significant gaps in screening and monitoring of bone health, suggesting the need to develop innovative strategies to overcome barriers to implementation, through policy, system, and infrastructure improvements.  The identified barriers were lack of time, structural support, training, and coordination among the healthcare team. |
| **36**  **Jones**  **2022** | **Canada**  A tertiary hospital  The largest cancer centre in Canada  Study date is not available | To implement and evaluate the impact of BoneRx on  1. Bone health care (BMD ordering, patient counselling);  2. patient engagement in HBB healthy bone behaviour,  3, patient knowledge and health beliefs regarding osteoporosis,  4. patient satisfaction | N = 292  (before n=143, after n=149, 86% recruitment)  Participant selection:  PCa exclude chemotherapy or metastasis  Understand English | Cross sectional Before – After cohort study  Patients questionnaire and chart review  Follow up at 6 months after ADT treatment | A prepopulated bone health prescription tool, entitled BoneRx, including BMD request and patient counselling of bone health, were provided at the initiation of ADT (point-of-care).    Patients were also provided with an educational booklet  Multiple enabling and reinforcing strategies were used based on the Awareness-to-Adherence and model of behaviour change | Before vs After  BMD test within 6 months of ADT: 34.7% vs 59.5%  Patient bone health counselling: 32.4% vs 59.9%  Vit D: 57% vs 81%  Calcium supplement: 39% vs 61%  Exercises: more engaged in moderate to vigorous activities  Osteoporosis knowledge, susceptibility, or health motivations: no difference  Patient satisfaction: 7.8/10 | BoneRx is a simple and acceptable strategy that is effective in increasing guideline adherent care and patient outcomes.  Need longer-term follow up to assess long-term maintenance of HBB in patients and clinical outcomes e.g. fractures  Need assess long-term sustainability  Research conducted in a single urban tertiary centre. No control. Reliance on self-report. |
| **37**  **Nadler**  **2013** | **Canada**  A tertiary hospital  June – Dec 2011 | To explore patients with PCa - ADT:  1. knowledge, self-efficacy (SE), and health beliefs about osteoporosis;  2 current engagements in HBBs.  3. the relationships between knowledge, SE, health beliefs, and engagement in HBBs. | **N = 175/330 (53% completion rate)**  Inclusion: current PCa receiving ADT injection;  Able to speak and read English  Exclusion: concurrent chemo or had metastasis  ADT duration: average 30months (1-221)  ADT <1 year 34%; ADT 1-5 years 39%; ADT>5 years 27% | **Questionnaire**  Completed in the clinic or sent it back by mail | Use theory of  Rosenstock’s Health Belief Model  Questionnaires included 4 sections:  1. Demographic,  2. Osteoporosis risk factors,  3. HBB assessment,  4. Validated measures for knowledge, SE, health beliefs.  DXA was requested if not received in the past 18 months. | **DXA scan in the past 2 years.**: 38%  **Osteopenia** (48%), **osteoporosis** (6%)  **FRAX**: moderate risk 21%, high risk 2%  **OP knowledge**: low  **Perceived SE**: moderate  **Health motivation**: fairly high  **Perceived susceptibility and seriousness of OP**: low.  **Vitamin D intake:** 42%  **Exercise**: 31%  **Calcium**: 15% were at risk of over-supplementation of calcium.  Patients taking calcium, vitamin D and for exercise had significantly greater knowledge than those who did not | Most patients who are receiving ADT are not receiving appropriate screening, lack basic information about bone health, and are not engaging in the appropriate HBBs. These findings support the application of the Health Belief Model in this population: Interventions that teach patients about the implications of bone loss, encourage proper uptake of HBBs, and promote feelings of SE could increase engagement in HBBs to prevent and manage bone loss.  The study suggests the need for active knowledge translation. |
| **38**  **Tsang**  **2018** | **Canada**  Tertiary cancer centre  Before:  2013-2014  After:  2014-2016 | To evaluate the ability of a multimodal patient education initiative to improve adherence to HBBs in men with PCa-ADT | **N=103**  N = 51 before, (recruitment 86%)  N = 52 after, (recruitment 72%)  I:nclusion  Received ADT < 12 months or plan to start ADT within 3 weeks for ADT > 6/12  Exclude:  Unable to exercise, severe cardiac disease, bone endocrinopathy, stage IV V CKD | Pilot prospective, single-site, before-after clinical trial  Patient self-report at regular 3 months follow up  Primary endpoints: feasibility of study methods and adherence to HBB  Secondary endpoints:  Receipt of BMD testing | Multimodal HBB education:  BoneRx (including DXA refer) +  Focused F2F education with an oncology nurse 60% or physician 40%  Patients were provided with customised educational materials. | Patients were satisfied with the study intervention, found educational materials easy to understand, and felt that it increased their knowledge about osteoporosis.  Intervention appeared to be associated with trends toward improved HBB, none of them were statistically significant.  Patients who received the study intervention were more likely to receive BMD testing (OR 3.3, 95% CI 1.3–8.8). | A brief tailored educational intervention was feasible to implement and improve BMD tests.  It did not increase HBB participation.  Changing patient behaviours may require more than a brief one-time educational intervention to be effective.  Future improvements included exploration of patient barriers and facilitators of life-style change.  The study was limited by the small number of participants. |
| **39**  **Zhumkhawala**  **2013** | **US**  Within a primary care population based membership programme  2003 – 2009 | To determine whether the implementation of the HBP (healthy Bone Program) screening and treatment protocol was effective in reducing the rate of osteoporotic hip fracture in men with PCa- ADT | **N =1482**  1071 intervention  411 control  Participants:  Newly diagnosed PCa, received leuprolide as monotherapy (first injection was index date)  Exclude if <50 years old, had DXA >3 month prior, previous hip fracture, only 1 injection, or <6 months f/u  Hip fractures exclude pathologic or traumatic fracture | **Retrospective** cohort  Electronic medical record system and cancer registry were the primary data source | Intervention: through an existing osteoporosis prevention program (HBP) which was to identify people at risk of osteoporosis and fracture and initiate DXA and treatment or refer to endocrinologist  Control: all others not in the programme | **Incidence of Hip fracture per 1000-person year**  Reduced by 70%. Intervention 5.1(3.0-8.0) vs Con 18.1 (10.5-29)  **Hip fracture mean interval between event date and first ADT injection**  Delayed in the Intervention 828.7 days vs Con 590.3 days  **Receive BPM treatment**  Intervention 29.2% vs Con 3.2%  **Hazard ratio (HR**)  Hip fracture in non-HBP: 4.19  Obese: 0.23  Number of ADT injection: 0.78 | Enrolment in an active screening and treatment of osteoporosis management programme effectively reduced hip #, avoiding this morbid complication of ADT |
